# 920. Difficult or Distressed? Reframing, Understanding, and Responding to Challenging patient and Family Interactions

**DOI:** 10.1093/jbcr/irag033.445

**Published:** 2026-04-06

**Authors:** Lindsey T Harris, Taylor McClendon, Carey K Lamphier, Pamela Vanderberg, Kristen Glasgow-Petela, Yuk Ming Liu, Laura S Johnson, Lauren B Nosanov

**Affiliations:** Walter L. Ingram Burn Center, Grady Memorial Hospital, Atlanta, GA; Grady Memorial Hospital, Atlanta, GA; Grady Memorial Hospital, Atlanta, GA; Grady Memorial Hospital, Atlanta, GA; Morehouse School of Medicine, Decatur, GA; Emory University School of Medicine, Atlanta, GA; Grady Memorial Hospital / Emory School of Medicine / Morehouse School of Medicine, Atlanta, GA; Emory University School of Medicine, Atlanta, GA

## Abstract

**Introduction:**

Challenging interactions may cause burn injured patients and their families to be labeled by healthcare providers as “difficult,” reflecting conscious and unconscious biases and underappreciation of underlying factors. Factors such as limited access to healthcare, financial strain, housing instability, and systemic inequities contribute to fear, grief, loss of control, and distrust of medical systems. “Difficult” behaviors often reflect underlying distress rather than intentional resistance. Understanding the root causes of these behaviors, while equipping staff with strategies to cope and respond effectively, is critical for improving patient care experiences and reducing staff burnout.

**Methods:**

An educational slide show was developed utilizing established developmental and psychosocial theories, including Erikson’s psychosocial stages, Piaget’s cognitive development theory, and Bronfenbrenner’s ecological systems theory, to identify themes related to the “why” behind difficult behaviors for pediatric and adult patients. Topics include definitions and indicators of “difficult” patients and families, developmental milestones, differences in pediatric versus adult presentations, and evidence-based coping strategies. Sessions will be delivered by a burn-specialized Certified Child Life Specialist and Licensed Professional Counselor to all burn center staff following administration of a pre-survey. Impact on knowledge and confidence will subsequently be measured in post-surveys.

**Results:**

Pre-survey responses have been received from 41 multidisciplinary team members. Most reported confidence in identifying common “difficult” characteristics (92%) and contributors to challenging behaviors (83%). Fewer participants indicated confidence in using de-escalation strategies (75%) or in having adequate coping tools and strategies (73%). Nearly all expressed a desire to learn more about how to manage challenging patient and family dynamics (95%).

**Conclusions:**

Initial positive anecdotal feedback reflects the intrinsic value of this planned educational initiative. Reframing difficult behaviors as expressions of distress shifts care from conflict-driven responses to compassion-based interactions. We anticipate that this education will foster resilience, strengthen therapeutic relationships, and reduce staff burnout.

**Applicability of Research to Practice:**

Application of developmental and ecological theories can provide insight into difficult behaviors, informing tailored strategies for de-escalation and coping. By addressing both patient and provider needs, healthcare teams can transform challenging encounters into opportunities for connection and healing.

**Funding for the study:**

N/A.

